# KINFix – A formalin-free non-commercial fixative optimized for histological, immunohistochemical and molecular analyses of neurosurgical tissue specimens 

**DOI:** 10.5414/NP300907

**Published:** 2015-11-02

**Authors:** Harald Stefanits, Michał Bieńkowski, Markus Galanski, Goran Mitulović, Thomas Ströbel, Ellen Gelpi, Teresa Ribalta, Helle Broholm, Christian Hartmann, Johan M. Kros, Matthias Preusser, Johannes A. Hainfellner

**Affiliations:** 1 Institute of Neurology,; 2 Department of Neurosurgery,; 3 Department of Medical and Chemical Laboratory Diagnostics,; 4 Department of Medicine I,; 5 Comprehensive Cancer Center, Medical University of Vienna, Austria,; 6 Department of Molecular Pathology and Neuropathology, Medical University of Lodz, Poland,; 7 Institute of Inorganic Chemistry, University of Vienna, Austria,; 8 Neurological Tissue Bank of the Biobanc-Hospital Clinic-Institut d’Investigacions Biomèdiques August Pi i Sunyer (IDIBAPS), Barcelona, Spain,; 9 Department of Pathology, Hospital Clínic de Barcelona, Barcelona, Spain,; 10 Neuropathology Laboratory, Copenhagen University Hospital, Copenhagen, Denmark,; 11 Department of Neuropathology, Ruprecht-Karls-Universität Heidelberg, Heidelberg, Germany (current address: Department of Neuropathology, Institute of Pathology, Medical University of Hannover, Hannover, Germany),; 12 Department of Pathology, Erasmus MC, Rotterdam, The Netherlands

**Keywords:** KINFix, RCL2, ethanol-based fixative, formalin substitute, nucleic acid preservation, proteomics

## Abstract

An optimal fixative should ideally combine the advantages of formalin fixation and freezing, allowing for good preservation of histology and molecular components, easy handling and storage, lack of toxicity, and low costs. Most of these criteria are fulfilled by ethanol-based solutions, and due to our good experience with the commercial RCL2 fixative, reflected by our published single-center trial, we initiated a multicenter ring trial. However, during its course, RCL2 was discontinued on the market. Therefore, we created our own agent, KINFix, composed of the same main constituents as RCL2, and employed it in our laboratory with similar results. Here we present our evaluation of the three fixatives formalin, RCL2, and KINFix from the perspective of histopathology as well as nucleic acid and protein analyses in comparison to fresh frozen tissues together with the multicenter ring trial data for RCL2. We observe that RCL2 and KINFix offer comparable histomorphology and superior template for molecular analyses than formalin. Moreover, KINFix as freely available fixative might overcome some of the difficulties related to the commercial agents. Therefore, we conclude that KINFix might be an attractive complement to formalin in tissue processing and advocate its use in neuropathological practice.

Supplemental material is available for free at: http://www.dustri.com Clinical Neuropathology, Vol. 35; January/February, 2016

## Introduction 

Normal buffered formalin (NBF, aqueous formaldehyde solution) has been maintaining its status of the universal tissue fixative for over 100 years, mostly due to the combination of its low price, wide availability, and handling ease with the possibility of tissue storage in the form of paraffin-embedded blocks at room temperature. Tissue fixation is achieved by “cross-linking” of proteins and nucleic acids via methylene bridges [1, 2, 3], preserving tissue morphology and enabling further processing with methods such as histochemistry and immunohistochemistry, although epitope masking may be a limitation and needs to be overcome with antigen retrieval techniques. Therefore, laboratory protocols and workflows are optimized for the use of formalin-fixed paraffin-embedded (FFPE) material and various diagnostic, prognostic, or predictive immunohistochemical biomarkers have been validated. Albeit limited by nucleic acid degradation/fragmentation and introduction of artificial mutations, the methods of DNA and RNA analysis in FFPE samples have also been established [3, 4, 5, 6]. On the other hand, formaldehyde is a harmful carcinogen [7], posing a risk to the personnel and requiring powerful ventilation devices and costly disposal procedures [8]. 

For this reason, the search for alternative fixatives continued over the last two decades, resulting in the introduction of several alcohol-based solutions (including FineFIX, RCL2, and PAXgene), which have been reported to conserve tissue morphology as well as proteins and nucleic acids comparably to NBF and fresh frozen tissue, respectively [1, 5, 9, 10, 11, 12, 13, 14]. Despite their applicability in the routine pathological practice without major protocol adaptations for most purposes and their lack of negative impact on antigen availability or nucleic acids with longer fixation times [3, 15], no alternative fixative has gained wide acceptance so far. Formalin fixation introduces specific artifacts into the tissue, which are often employed by the pathologists to formulate a diagnosis [8, 15, 16, 17, 18, 19]. The artifacts created by the alternative fixatives are slightly different (such as erythrocyte lysis, accentuation of nuclear details and increased eosinophilia) [3], which should not hinder the pathological diagnosis in most, but may cause uncertainty in borderline cases. Also, biomarkers have been validated for FFPE samples only. For these reasons, some adaptations both to laboratory protocols/workflows and to the training of pathologists are still necessary. Additionally, the effects of long-term storage and the optimal storage conditions have not been well documented yet. Finally, the commercial distribution of the alternative fixatives pertains to 3 major concerns: first, their acquisition costs are 3- to 10-fold higher in comparison to formalin [9]; second, the products may be discontinued by the producers at any time (as it happened in the case of RCL2) [8]; third, the undisclosed recipes create uncertainty whether long-term archiving can be guaranteed for the future. 

Therefore, we would like to present KINFix, a novel ethanol-based fixative, which is similar to RCL2 and provides both conservation of tissue morphology and preservation of proteins and nucleic acids (including genomic DNA and mRNA). Hereby, we fully disclose the recipe and the requirements for its introduction into the neuropathological workflows, which will allow for its easy production and application in every laboratory. A freely available alcohol-based fixative may be an attractive complement for formalin, especially in laboratories without the large storage facilities for frozen tissue or in cases that small biopsy specimens require both histological examination and molecular analyses of prognostic or predictive markers. 

## Materials and methods 

### KINFix recipe for in-house production 

We developed KINFix based on the published data concerning ingredients of commercially and freely available alternative fixatives. The term KINFix derives from “Klinisches Institut für Neurologie Fixative”. For the working solution, add 537 mL of acetic acid to 2,000 mL ddH_2_O (**Cave**, do not pour ddH_2_O into the acetic acid!). Fill up to 3,000 mL of volume with ddH2O. Add 480 g of trehalose. Dilute with 5,000 mL of 100% ethanol. The result is 8,000 mL of KINFix ready-to-use working solution (Table 1). The ingredients for 1 liter of KINFix working solution cost ~ 75 € compared to 1.10 € for NBF (Table 1). The in-house produced ready-to-use working solution was analyzed in terms of chemical composition after the mixture of the individual ingredients and stability over time (see below). KINFix working solution can be stored at +4 °C for ~ 120 days. Crystallized sugar may precipitate, guaranteeing a steady concentration in the solution. 

### Tissue fixation with KINFix 

In our laboratory, tissue is dissected into pieces of 20 × 20 × 5 mm maximum, and embedded into plastic cassettes; the size of the specimen should not exceed 50% of the cassette inner volume. Fixation by immersion can be achieved with an approximate amount of at least the 5-fold volume of tissue specimen for 24 – 48 hours at +4 °C (i.e. 20 – 50 mL of KINFix working solution per specimen). Afterwards, the specimen is dehydrated in 100% ethanol 3 × 50 minutes at +4 °C, 3 × 50 minutes at room temperature, xylene for 45 and 60 minutes, and then embedded in low-melting paraffin (52 – 56 °C) for 3 × 110 minutes. The blocks may be stored at room temperature; however, if KINFix is used as an alternative solution to freezing, storage at –20 °C is advised. 

### Chemical analyses 

Working solutions of KINFix and an expired batch of RCL2 (Alphelys, France) were investigated by ^1^H and ^13^C nuclear magnetic resonance (NMR) spectroscopy to determine chemical similarities between both fixatives (see Supplement for details). Experiments were repeated after 3 months of storage in order to detect changes in chemical stability. 

### Histopathology 

Comparison of tissue morphology as well as immunohistochemical stains were performed in-house. Seven cases of neurosurgical biopsy specimens were prepared as published by Preusser et al. [14]: briefly, hematoxylin-eosin (H&E) and histochemical (Gomori-Trichrome, Alcian blue, Periodic acid Schiff) stains were performed following the same protocol for all three fixatives. Protocols for immunohistochemistry were adapted in most antibodies for the ethanol-based fixatives. Immunohistochemical stains with markers used in routine surgical neuropathological diagnostics (GFAP, S-100, Vimentin, EMA, Synaptophysin, NeuN, Map-2, SMI31, SMI32, NSE, Pan-CK, Ki-67, p53, EGFR, Olig2, CD34) were performed on an autostainer (Dako, Glostrup, Denmark) with a standard incubation time of 25 minutes at room temperature (see Table S2 for an overview of antibodies and the respective protocols). ATRX was stained manually using the coverplate method incubating the primary antibody over night at +4 °C. Pretreatment consisted of either incubation with Target Retrieval Solution pH6 (TRS low, Dako) or Target Retrieval Solution pH9 (TRS high, Dako) for 20 minutes in the pretreatment-module of the autostainer system, or Proteinase K ready-to-use treatment for 5 minutes at room temperature (ProtK rtu). Visualization of the primary antibody was highlighted by the Flex+ mouse/rabbit detection system (Dako). 

### Nucleic acid and protein analyses 

For the analyses of nucleic acids and protein composition, formalin-fixed paraffin-embedded (FFPE), RCL2-fixed paraffin-embedded (RCLPE), KINFix-fixed paraffin-embedded (KFPE), and fresh frozen (FF) material from four tissue samples was evaluated. Samples included two neocortical specimens of patients with temporal lobe epilepsy (N702-12, N882-12), one specimen of pilocytic astrocytoma (N748-12), and one specimen of glioblastoma (N886-12), the proteomic analysis was additionally performed for one specimen of diffuse astrocytoma (N852-12). From all samples, genomic DNA and total cellular RNA were isolated, and RNA was reverse transcribed into single stranded cDNA (see Supplement for details). To assess the quality of the isolated nucleic acids, a panel of PCR reactions was performed for each DNA/cDNA sample and the products were separated on 1% agarose gel and visualized (see Supplement for detail). For the proteomic analysis, protein was extracted from all samples and HPLC-MS/MS analysis was performed (see Supplement for details). 

### Ring trial 

To compare the usefulness of ethanol-based fixatives for diagnostic purposes, we performed a ring trial focusing on the neuropathological evaluation of brain tumor specimens fixed with RCL2 and formalin according to criteria of the WHO classification of tumors of the central nervous system. 15 different primary brain tumor samples (2 meningothelial meningiomas, 2 malignant meningiomas, 1 anaplastic ependymoma, 2 diffuse astrocytomas, 1 pilocytic astrocytoma, 1 anaplastic astrocytoma, 1 ETANTR, 1 glioblastoma with oligodendroglial features, 1 glioblastoma, 1 anaplastic oligo-astrocytoma, 1 ependymoma, and 1 medulloblastoma) as well as 1 carcinoma metastasis were analyzed by four raters from different European institutions (H.B., E.G., C.H., J.M.K.). Four to 9 characteristic features of each tumor (86 features in total) were evaluated using a pre-defined questionnaire (Tables S7, S8). 

## Results 

### Chemical analyses and comparison with RCL2 

The NMR spectra of freshly prepared KINFix showed signals of trehalose, acetic acid, and ethanol (in correspondence to the components used) as well as small amounts of ethyl acetate (Figures S9, S10). The results obtained for the working solution prepared with the expired batch of RCL2 were similar in principle (Figures S6, S7), however, α- and β-Glucose were additionally detected and the concentration of ethyl acetate was different (see Supplement for details). 

The stability of KINFix was assessed over a period of 3 months. The NMR spectra showed an increase in the amount of ethyl acetate, but no additional sugar resonances were detected, suggesting that the disaccharide was not split under these conditions in contrast to RCL2 (see Supplement for details). The NMR spectra for the air-dried precipitate showed the signals for trehalose only, thus, after purification, it may be used for the preparation of further batches of KINFix (see Supplement for details). 

### Integration of KINFix into daily laboratory routine 

We started to integrate the alternative fixative RCL2 into our laboratory routine in 2008 which was succeeded by KINFix after the discontinuation of RCL2 in 2012. The embedding protocol for KINFix differs from the protocol used for NBF, thus, an additional equipment for automated dehydration and paraffin-embedding had to be acquired in order to process both fixatives in parallel. KINFix handling is similar to formalin, which met good acceptance from our technicians. Importantly, no changes to the archival system had to be applied and only minor adaptations of tissue database software had to be done. 

### Histopathological and immunohistochemical staining 

**Figure 1.**
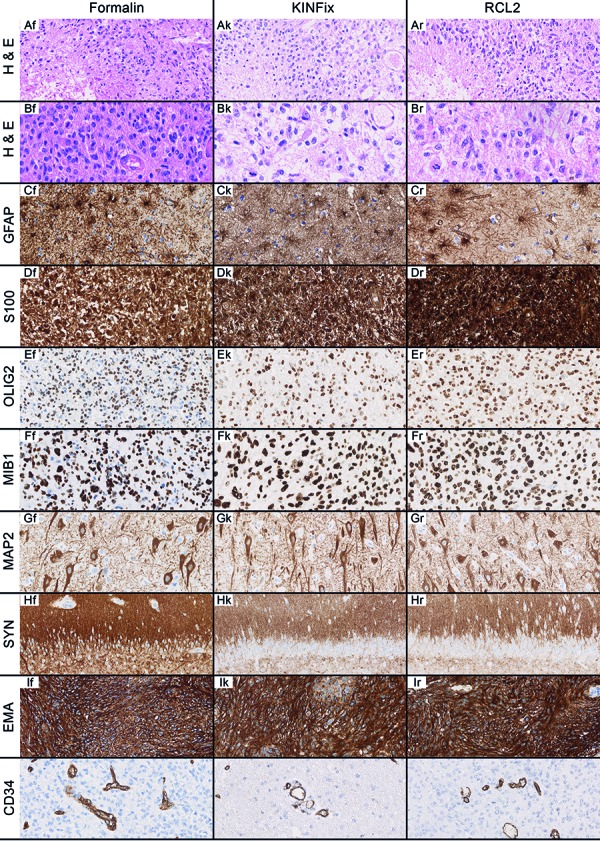
Histological and immunohistochemical stains of FFPE- (f), KFPE- (k), and RCLPE- (r) fixed tissue specimens: (A, B, D, E) glioblastoma multiforme; (C, J) diffuse astrocytoma; (F) sarcoma; (G, H) hippocampus (epilepsy surgery); (I) atypical meningioma; 400 × magnification, B in 800 × magnification.

Over a time period of 7 years, H&E stains were made for both FFPE and RCLPE/KFPE tissue specimens of almost all histopathological specimens in our laboratory; equivalent performance was observed for both alcohol-based fixatives. To verify this impression, we compared H & E and immunohistochemically stained sections of FFPE, RCLPE, and KFPE tissue samples in analogy to the analysis conducted by Preusser et al. (Figure 1) [14]. In general, KINFix performed equal to RCL2 and comparable to FFPE, however, for some antibodies, protocols had to be adapted in terms of antigen retrieval and antibody dilution (see Table S2 for details). Only two antibodies out of the panel of tested antibodies, i.e., anti-BAF47 and anti-CK-Lu5 (pan-CK), did not work in RCL2 and KINFix-fixed tissue. 

### Nucleic acid and protein analyses 

**Figure 2.**
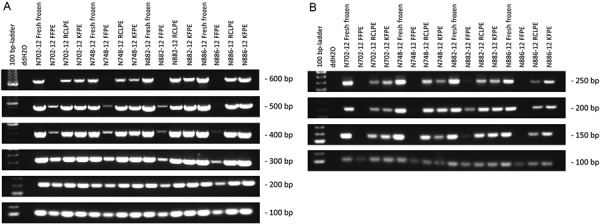
A: PCR products from DNA isolated from FFPE-, KFPE-, and RCLPE-fixed tissue and frozen samples. The fragments up to 600 bp may be amplified with good efficiency from ethanol-based fixatives and fresh frozen material. B: PCR products from RNA (cDNA) isolated from FFPE-, KFPE-, and RCLPE-fixed tissue and frozen samples. The fragments up to 250 bp may be amplified from fresh frozen and KFPE-/RCLPE-fixed material.

DNA isolated from frozen, RCLPE, and KFPE material could be efficiently amplified up to 600 bp, while FFPE-derived template yielded decreasing amounts of PCR product with its complete lack at 600 bp (Figure 2A). Similarly, frozen, RCLPE, and KFPE material allowed for the amplification of cDNA up to 250 bp, while FFPE-derived template yielded almost no PCR product larger than 100 bp (Figure 2B). 

Proteomic analyses revealed high protein yields for all samples and fixatives, which identified between 95 and 3,074 proteins. There was no statistically significant difference between the fixatives in terms of total and exclusive protein yield for each fixative (see Figure S14 for details). 

### Diagnostic performance of alcohol-based fixatives compared to formalin 

In a pan-European ring trial, we evaluated the applicability of RCL2 as a fixative for routine neuropathological diagnostics. Overall, 86 features of brain tumor specimens were evaluated by 4 raters in 16 different specimens (344 feature and 64 diagnostic applicability assessments in total). 

In total, 323 features (93.9%) were reported as present and none as absent in both fixatives. While present in the other sample, 9 (2.6%) features were marked as absent (4 as “indeterminate” –1.1%) in RCLPE and 2 (0.6%) were not seen in FFPE (3 marked as “indeterminate” – 0.9%). One feature (mitotic figures in medulloblastoma) was marked by one rater as “indeterminate” in both samples. 

For the comparative assessment, both fixatives were evaluated as equal in 184 cases (53.5%), FFPE samples performed better in 71 (20.6%), while RCL in 81 cases (23.5%); no conclusion was reached for 8 cases (2.3%). 

Finally, 50 times RCLPE was marked to not compromise the neuropathological diagnosis, while 12 and 2 times a compromising and indeterminate score, respectively, was assigned. Combining, 5 and 4 cases were marked as compromised in RCLPE by 2/4 and 1/4 raters, respectively; while 7 cases were unanimously marked as not compromised (for descriptive evaluation see Table S8). 

## Discussion 

Formalin is the universal tissue fixative, in particular due to excellent preservation of morphological details and the possibility of long-term storage of samples at room temperature. Over time, immunohistochemical and DNA/RNA analyses have been adapted, and with antigen retrieval techniques and special nucleic acid extraction kits [3] some of the disadvantages of formalin fixation could be overcome [2]. Novel advanced technologies, requiring high quality of protein and nucleic acids, have been introduced into clinical (particularly oncological) practice and offer relevant insight into the pathogenesis and prognostic/predictive factors for an individual patient [20, 21, 22]. Fresh frozen tissue is the gold standard for such purposes, however, the preparation and storage of samples at –70 °C is elaborate, expensive and not always possible (e.g., small biopsy specimens cannot be split into two parts, and professional laboratory staff is usually not available in the evening and on weekends). Furthermore, the destruction of fine tissue morphology significantly decreases the spatial resolution, while the degradation of nucleic acids cannot be entirely avoided at long-term storage [23]. 

An optimal fixative would combine the advantages, while minimizing the flaws, of formalin and freezing, i.e., easy handling and storage without toxicity, low costs, good gross and microscopic morphology as well as preservation of proteins and nucleic acids. The search for such a fixative has continued since the 1980s and most of these criteria are fulfilled by ethanol-based solutions. They act via protein coagulation, which is also employed in formalin fixation at the dehydration step [3]. The addition of acetic acid (as in KINFix) protects from alcohol-induced tissue shrinkage [3], while polyethylene glycol (PEG, e.g., in Kryofix or Boonfix) causes further dehydration, protecting from protein and DNA degradation [3]. The common features include rapid fixation, greater stain avidity and the lesser demand for antigen retrieval techniques, however, at the cost of nuclear shrinkage and increased variability of tissue staining as well as artificial pigment deposition in bloody specimens and slightly increased viscosity. The increased flammability is outweighed by the easy disposal and elimination of carcinogenic vapors [3]. Nevertheless, the prefixation factors (such as warm and cold ischemia time at surgery, transport conditions, speed of fixative penetration, thickness of tissue blocks, type of tissue, processing protocols or storage conditions) affect the specimens independently of the type of fixative (ethanol-based and formalin) [3, 5, 24, 25]. 

In general, the experience with FFPE samples allows for the diagnostic use of H & E stains after ethanol-based fixation [3], as was shown for FineFIX, RCL2 [26], HOPE [12], and PAXgene [16, 27] and which is in accordance with our observations of RCL2 and KINFix as well as with our RCL2 multicenter ring trial. In this ring trial, in over 75% of cases RCL2 was regarded as equally or better applicable for diagnostics in comparison to formalin, whose superiority in 20.6% might result from slightly different and accustomed to artifacts [3, 4, 9, 16, 28], which could be further diminished with additional training along a new training curve [3, 16, 17, 18]. What is important, alcohol-based fixation is well applicable for IHC due to its high standardizability (no “overfixation”) [3, 15, 16, 29], lack of cross-linking [4] (and thus, epitope masking [9, 14, 16, 30, 31]) and lower requirement for antibody concentration [12, 13, 29] as well as possible use of many antibodies which are not applicable to formalin-fixed tissues [12, 15, 23, 26, 29]. However, all markers have to be re-evaluated for sensitivity and specificity [8, 9, 12, 13, 16, 26, 28, 32, 33]. Similarly, alcohol-based fixatives often show superiority in both classical (e.g., western blot) and modern proteomic analyses (nano LC-ESI-mass spectrometry, MALDI-MS) [12, 13, 23, 34, 35], which was also observed in our study. Furthermore, alcohol-based fixatives may provide the appropriate material (comparably to fresh or frozen tissue [4, 36, 37]) for biomarker analysis with novel methods requiring high quality (e.g., whole genome amplification [25, 34]) or quantity (e.g., methylation arrays [25, 38]) of DNA. Although some methods are compatible with FFPE samples (e.g., aCGH or SNP array), a better template, offered by the ethanol-based fixative [6, 10, 14, 26, 27, 28, 39, 40], is always beneficial. The formalin-induced artificial mutations might also be mistaken for genuine findings and have several times been incorporated into databases [3, 36, 41]. Ethanol-based fixatives especially excel at expression profiling of microdissected areas, which entirely depends on the combination of excellent morphology and RNA preservation [42] and which is feasible neither with formalin nor with frozen tissue [2, 3, 4, 5, 39]. In general, alcohol-based fixatives were shown to preserve RNA comparably to frozen tissue and significantly better than formalin [2, 4, 24, 27, 28, 32, 43] (except for short RNAs, e.g., miRNA, which may be adequately analyzed from FFPE material [1, 3, 25]). Still, currently ethanol-based fixatives are the only alternative to double sampling if analysis of both morphology and longer RNA fragments is required [44]. All the issues brought up here in this paragraph in brief are more extensively discussed in the Supplement. 

Our experience with ethanol-based fixatives has been gathered since 2008, when we introduced RCL2 into routine histopathology (as a second fixative parallel to formalin for each specimen) and this project was initially aimed as an assessment of its diagnostic performance. Unfortunately, before it was finished, the production of RCL2 had been discontinued (in 2012) rendering the concept obsolete. In line with the need for a successor of this fixative, we decided to attempt at creating our own, similar agent. Starting with the basic description of RCL2 and of other ethanol-based fixatives as well as with the data from NMR analysis, we developed KINFix and reintroduced the parallel fixation. Based on 3 years’ experience we conclude that the results obtained with RCL2 and KINFix are highly similar. Nevertheless, both agents cannot and should not be considered to be the same, as the detailed recipe of RCL2 has never been fully disclosed by the manufacturer. We believe that a freely available ethanol-based fixative, as we have it now with KINFix, may overcome some crucial obstacles for a novel fixative, like costs and accessibility. 

To conclude, the freely available alcohol-based fixative KINFix provides excellent histological morphology as well as preserved proteins and nucleic acids. Properties of KINFix, e.g., long-term preservation of histology and molecules in paraffin-embedded specimens, require continued observation and evaluation. Multicenter international ring trials evaluating characteristic artifacts, biomolecule stability during long-term storage, storage conditions, protocol optimization, and reliability in pathological diagnostics are to be conducted. Such studies, in frame of a pan-European consortium (SPIDIA), have already been initiated; however, a wide acceptance of one fixative is necessary. In our opinion, only a freely available (and not commercially marketed) fixative can succeed in complementing formalin-fixed and fresh frozen tissue. 

## Conflict of interest 

The authors report no conflict of interest. 

## Acknowledgments 

We acknowledge the excellent technical assistance of Elisabeth Dirnberger. Michał Bieńkowski was supported by the Healthy Ageing Research Centre project (REGPOT-2012-2013-1, 7FP). 

**Table 1.** KINFix components. Costs of the aliquots used for 8,000 mL of working solution (based on the price of the product used in our laboratory). Hazard statements according to Globally Harmonized System of Classification and Labelling of Chemicals.

Ingredient	Amount	Costs	Product	Storage	Hazard statements
ddH_2_O	2,463 mL	N/A	In-house production	RT	None
Acetic acid 100%	537 mL	21.40 €	VWR, BDH, Prolabo Chemicals #20104.298	RT	H226, H303, H312, H314, H317, H331, H402
Trehalose	480 g	471.40 €	Roth #5151.3	RT	H303
Ethanol 100%	5,000 mL	106.80 €	VWR, BDH, Prolabo Chemicals #20821.330	RT	H225, H315, H320, H335, H401
Total	8,000 mL	599.60 €			

RT = room temperature.
